# The RyR-like-FKBP12-PKA Complex Regulates Intracellular Ca^2+^, Unfolded Protein Response and Apoptosis in *Patinopecten yessoensis* Under High-Temperature Stress

**DOI:** 10.3390/ijms27135859

**Published:** 2026-06-29

**Authors:** Wenfei Gu, Qingyu Peng, Chuanyan Yang, Hongbo Lu, Dongli Jiang, Lingling Wang, Linsheng Song

**Affiliations:** 1 Liaoning Key Laboratory of Marine Animal Immunology & Disease Control, Dalian Ocean University, Dalian 116023, China; 2 Dalian Key Laboratory of Aquatic Animal Disease Prevention and Control, Dalian Ocean University, Dalian 116023, China

**Keywords:** *Patinopecten yessoensis*, Ryanodine receptor-like, high-temperature stress, unfolded protein response, apoptosis

## Abstract

Ryanodine receptor-like (RyR-like) is a key endoplasmic reticulum (ER) Ca^2+^ release channel governing intracellular Ca^2+^ homeostasis and cellular stress responses in invertebrates. However, its function in bivalves under high-temperature stress remains unclear. In the present study, one RyR-like was identified from Yesso scallop *Patinopecten yessoensis* (*Py*RyR-like). Its function in regulating intracellular Ca^2+^, IRE1α-mediated unfolded protein response (UPR) and apoptosis in the mantle after high-temperature (25 °C) treatment was investigated using molecular cloning, qRT-PCR, Western blot, pull-down assay, cellular calcium imaging, TUNEL and histology assays; High temperature treatment significantly increased intracellular Ca^2+^ content at 1 and 6 h (*p* < 0.05), but decreased it at 3, 12 and 24 h (*p* < 0.05); meanwhile, the cAMP level, *Py*PKA activity, mRNA expression level of *Py*RyR-like, and protein expression levels of *Py*FKBP12 and *Py*GRP78 were significantly increased at different times. However, high temperature did not affect the expression level of *Py*NVL and *Py*XBP1(S). The SPRY and RYR domains of *Py*RyR-like separately interacted with *Py*FKBP12 and *Py*PKA. Moreover, RyR antagonist Dantrolene reversed high-temperature-induced alterations in Ca concentration, PKA activity, and core UPR- and apoptosis-related molecules, and suppressed Caspase-3 activity. These findings suggest that *Py*RyR-like plays an important role in the high-temperature response of scallops by regulating intracellular Ca^2+^ homeostasis and mediating UPR activation and apoptosis, providing new insight into the molecular mechanism underlying scallop adaptation to high temperature.

## 1. Introduction

Ca^2+^ is a second messenger of cells, and its homeostasis in the cellular environment plays an important role in maintaining various life activities [1]. The endoplasmic reticulum (ER) is the most important calcium reservoir within cells, and its Ca^2+^ content is controlled by the Ca^2+^ transport system, mainly through various Ca^2+^ channels, such as the Ryanodine receptor (RyR). RyR is a critical class of Ca^2+^ release channels located in the ER membrane. It regulates calcium homeostasis by releasing Ca^2+^ from the ER to the cytoplasm and plays an essential role in the maintenance of cellular excitability and physiological functions [2,3]. RyR is expressed in a variety of tissues, with the highest density observed in striated muscle [4,5]. To date, three subtypes of RyR have been identified in *Homo sapiens*, namely RyR1, RyR2 and RyR3. Their expression exhibits tissue specificity in striated muscles. RyR2 is the predominant isotype in the myocardium, where it mediates Ca^2+^ release from the sarcoplasmic reticulum (SR), the specialized ER in cardiomyocytes, thereby modulating cardiac contractile force [6].

Studies in mammals indicate that RyR2 plays vital roles in regulating Ca^2+^ homeostasis and the ER stress response. Human RyR2 comprises four MIR domains, two RYDR_ITPR domains, three SPRY domains, four RYR domains, one RIH_assoc domain, two EFh domains, one RR_TM4-6 domain and one Ion_trans domain. SPRY1 and RYRs, containing binding sites for the small molecule FKBP12 and protein kinase A (PKA), respectively, are critical domains for stabilizing the channel and regulating channel opening [7,8]. PKA phosphorylation dissociates FKBP12.6 from RyR2 and regulates the opening probability of RyR2 channels [9]. PKA also mediates increased phosphorylation of RyR2, leading to increased sensitivity to Ca^2+^-induced activation, which in turn leads to impaired channel function [9,10]. Moreover, the phosphorylation degree of RyR2 is highly correlated with the extent of calcium leakage [11]. It has been reported that calcium sparks in human muscle cells are significantly enhanced in terms of spatiotemporal patterns due to the increased activity of the RyR2 channel [12]. The latest research shows that the differential effect of RyR2 on and off rates determines the SR Ca^2+^ threshold for Ca^2+^ phenomena induced by Ca^2+^ embers, waves, and storage overloads [13]. Once activated, RyR2 rapidly releases a large amount of Ca^2+^ into the cytoplasm, leading to a sharp increase in cytoplasmic calcium and inducing ER stress. It has been reported that arsenic (As) and antimony (Sb) stimulation significantly increased the expression levels of the RyR2 gene, unfolded protein response (UPR)-related genes and apoptosis-related genes in mice hearts, triggering the ER apoptotic pathway [14]. Furthermore, downregulating RyR2 increases the oxygen consumption rate; reduces the expression of GRP78, ATF3 and ATF6; and reduces oxidative stress [15]. Another study in cardiomyocytes indicated that RyR2 dysfunction activates the UPR primarily through ATF4 activation to further regulate genes related to protein biosynthesis [16]. Therefore, further work is still needed to elucidate the mechanism by which RyR regulates ERS.

Compared with the in-depth research in mammals, the studies on RyRs in aquatic animals remain at an early stage and have mainly focused on their gene expression and Ca^2+^ regulation in fish. Several studies have shown that the expression of RyRs in fish shows species and tissue differences. For example, the expression level of RyRs is higher in cold-acclimated (4 °C) trout in burbot and crucian carp [17]. In addition, RyRs in fish muscle and heart cells are present at a lower density than mammalian RyR2 channels and exhibit greater spatial separation within the SR membrane [18,19]. The relative expression of RyR genes in *Pimephales promelas* was elevated following exposure to the compound IMI and CHL [20]. Similar to their roles in mammals, aquatic RyRs also play a crucial role in calcium signaling. RyR has been reported to regulate Ca^2+^ in the presynaptic nerves of *Aplysia californica* [21] and in the embryonic skeletal muscle of zebrafish [22], while RyRs in fish hearts exhibit reduced sensitivity to Ca^2+^ release [17,18,19]. Moreover, RyR-mediated release of Ca^2+^ from the ER may lead to dysregulation of lipid metabolism in yellow catfish [23] and cell cycle arrest in *Chlamys farreri* [24]. However, the precise mechanism through which RyR facilitates Ca^2+^ release in aquatic animals under high-temperature stress remains unclear. In particular, compared with vertebrates, the role of RyR-like-related Ca^2+^ regulation in mollusks remains poorly understood. Whether RyR-like participates in high-temperature-induced Ca^2+^ disturbance and UPR-related responses in bivalves, and whether FKBP12 and PKA are involved in this process, remain unclear.

Yesso scallops (*Patinopecten yessoensis*) are cold-water shellfish that thrive at water temperatures ranging from 10 to 15 °C. However, continuous global warming has threatened the survival of this scallop and has even led to mass mortality [25]. Given that the maintenance of calcium homeostasis is essential for both cell survival and apoptosis, the present study identified RyR, one of the most important calcium release channel proteins on the ER membrane, from the scallop *P. yessoensis* and investigated its potential role under high-temperature stress. The objectives of this study were to: (1) analyze the expression pattern and structural characteristics of *Py*RyR-like; (2) examine its potential involvement in regulating cytoplasmic and ER Ca^2+^ signals; and (3) explore the relationship among *Py*RyR-like-related Ca^2+^ regulation, UPR-related responses and apoptosis following high-temperature treatment. Unlike previous studies that mainly focused on RyR-related Ca^2+^ regulation in vertebrates, this study provides new insight into the possible role of a RyR-related regulatory axis in the high-temperature response of a marine bivalve. These findings will provide a foundational basis for further research into the response mechanisms and calcium homeostasis of scallops under high-temperature stress.

## 2. Results

### 2.1. PyRyR-like Includes Classical Domains but Exhibits Low Sequence Conservation

**Figure 1:**
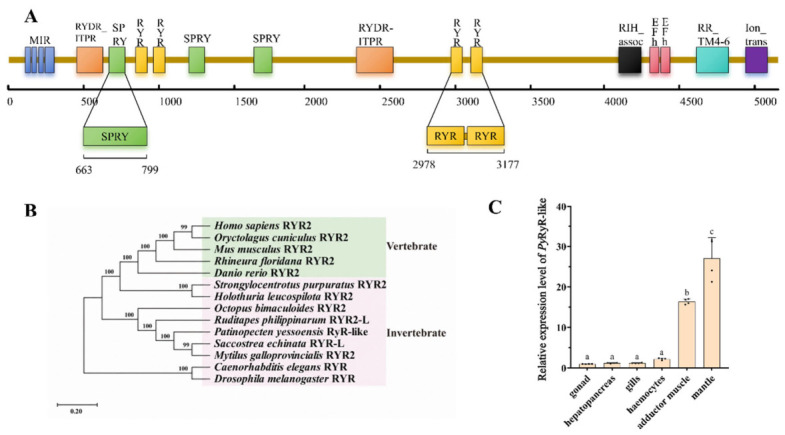
The domain architecture, phylogenetic tree and tissue distribution of *Py*RyR-like: (**A**) Domain architecture of *Py*RyR-like. (**B**) Phylogenetic tree of 14 RyR-likes from various species (Table 2). (**C**) The relative expression level of *Py*RyR-like mRNA in different tissues and hemocytes. The distinct letters (a, b, and c) were used to indicate the significant differences. Each value is shown as mean ± S.D. (N = 4).

The length of *Py*RyR-like polypeptide is 5191 amino acids, and consists four MIR domains (101–155, 162–204, 214–268, and 274–364 aa), two RYDR_ITPR domains (444–641 and 2338–2585 aa), three SPRY domains (663–799, 1092–1215, and 1644–1790 aa), four RyR domains (851–941, 966–1056, 2978–3068, and 3093–3177 aa), one RIH_assoc domain (4127–4245 aa), two EF-hand domains (4324–4352 and 4359–4387 aa), one RR_TM4-6 domain (4638–4843 aa), and one Ion_trans domain (4949–5101 aa) (Figure 1A). Notably, the sequences of the SPRY1 and RYR3-4 domains of the RyR-like proteins are highly conserved across species showed a high degree of consistency. Among them, the RYR3-4 domain is a phosphorylation hotspot domain involved in regulatory interactions and may provide a structural basis for the PKA-mediated regulation of channel activity during the heat stress response. There are multiple binding sites on the SPRY1 domain and the RYR3-4 domain for *Py*FKBP12 and *Py*PKA, respectively, which control the opening of the pore (Figure 1A).

Multiple sequence alignment revealed that the deduced amino acid sequence of *Py*RyR-like shared low similarity with RyR2s sequences from other organisms, including *Homo sapiens* (NP_001026.2, 46.6%), *Mus musculus* (NP_076357.2, 46.47%), *Danio rerio* (XP_068080773.1, 45.3%), *Drosophila melanogaster* (NP_001246209.1, 49.73%), *Caenorhabditis elegans* (NP_001343688.1, 42.58%), *Holothuria leucospilot*a (KAJ8044966.1, 43.24%), and *Strongylocentrotus purpuratus* (XP_030855111.1, 45.57%), while it shared high similarity with RyR-likes from *Octopus bimaculoides* (XP_052827807.1, 65.18%), *Ruditapes philippinarum* (XP_060571016.1, 69.46%) and *Saccostrea echinata* (XP_061163617.1, 71.59%) (Figure S1).

Fourteen RyR2s from different species were clustered into two groups, the vertebrate RyR2s and the invertebrate RyR2s. *Py*RyR-like was first clustered with RyR2s from other molluscs (*Mytilus galloprovincialis* RyR2, *Saccostrea echinata* RyR2, *Ruditapes philippinarum* RyR2 and *Octopus bimaculoides* RyR2), and then gathered with the RyR2s from echinodermatas (*Holothuria leucospilota* RyR2 and *Strongylocentrotus purpuratus* RyR2) and vertebrates (*Homo sapiens* RyR2, *Mus musculus* RyR2, *Danio rerio* RyR2, *Oryctolagus cuniculus* RyR2, *Rhineura floridana* RyR2), and finally gathered with the RyR2s from *Caenorhabditis elegans* and *Drosophila melanogaster* (Figure 1B).

The results in Section 2.1 indicate that *Py*RyR-like exhibited a certain degree of conservation and variability, which may be associated with its classical role and distinct functions compared to those in high animals.

### 2.2. PyRyR-like Is Widely Expressed in Scallops with the Highest Expression in Mantle

*Py*RyR-like transcripts were detected in the mantle, gonad, gill, adductor muscle, hepatopancreas, and hemocytes, with the highest expression level observed in the mantle. The expression level in the mantle was 26.99-fold higher than that in the gonad (*p* < 0.05). Furthermore, the expression level in the adductor muscle was 16.37-fold higher than that in the gonad (*p* < 0.05) (Figure 1C).

The results in Section 2.2 indicated that *Py*RyR-like may be involved in essential physiological processes in scallops, and its highest expression in the mantle suggested an important role in temperature sensing and environmental stress adaptation.

### 2.3. Inhibition of PyRyR-like Attenuates High-Temperature-Induced Ire1-Mediated Upr and Apoptosis by Reducing Intracellular Ca^2+^

#### 2.3.1. High Temperature Increases Camp Level, *Py*PKA Activity, *Py*RyR-like/*Py*FKBP12 Expression, and Intracellular Ca^2+^ Level in Mantle

**Figure 2:**
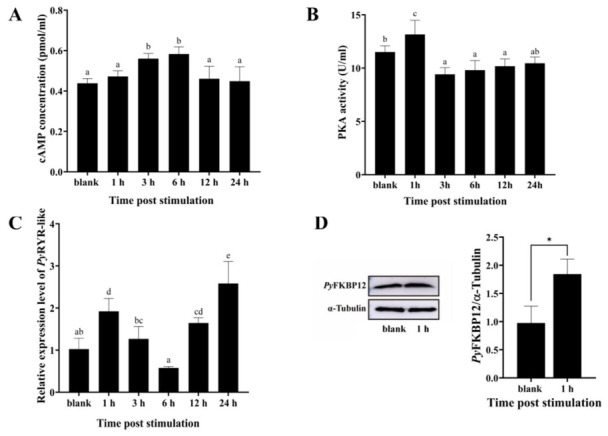
Changes in cAMP concentration, PKA activity, *Py*RyR-like and *Py*FKBP12 expression level in mantle after high-temperature treatment: (**A**) cAMP concentration. (**B**) PKA activity. (**C**) Relative expression level of *Py*RyR-like mRNA. (**D**) Western blot analysis of *Py*FKBP12 protein. Distinct letters (a, b, c, d and e) were used to indicate the significant differences. Each value is shown as the mean ± Standard deviation (S.D.) (N = 4). * *p* < 0.05.

After 3 and 6 h of high-temperature treatment, the cAMP levels in the mantle increased significantly, reaching 1.28- and 1.33-fold of that in the blank group, before returning to normal levels (*p* < 0.05) (Figure 2A). *Py*PKA activity in the mantle was markedly elevated at 1 h after treatment, showing a 1.14-fold increase compared to the blank group (*p* < 0.05) (Figure 2B). The relative expression of *Py*RyR-like mRNA exhibited a significant rise at 1 h (1.88-fold of that in the blank group), and peaked at 24 h (2.53-fold of that in the blank group) (*p* < 0.05) (Figure 2C). Following one hour of high-temperature treatment, the protein expression level of *Py*FKBP12 was increased significantly, reaching 1.26-fold of that in the blank group (*p* < 0.05) (Figure 2D).

**Figure 3:**
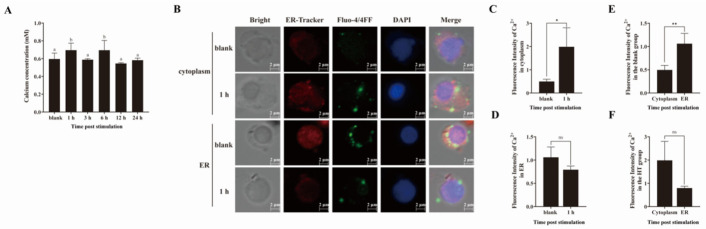
The alteration in Ca^2+^ concentration and distribution in the cytoplasm and ER of the mantle after high-temperature treatment: (**A**) The intracellular Ca^2+^ content. (**B**) The Ca^2+^ distribution. (**C**) The mean fluorescence intensity of Ca^2+^ in the cytoplasm. (**D**) The mean fluorescence intensity of Ca^2+^ in the ER. (**E**) The mean fluorescence intensity of Ca^2+^ in the cytoplasm and the ER in the blank group. (**F**) The mean fluorescence intensity of Ca^2+^ in the cytoplasm and the ER in the HT group. Distinct letters (a, and b) were used to indicate the significant differences. Each value is shown as mean ± S.D. (N = 4). * *p* < 0.05; ** *p* < 0.01; ns, not significant.

After high-temperature treatment, the concentration of Ca^2+^ in the mantle was significantly elevated at 1 and 6 h, reaching 1.17- and 1.16-fold of that in the blank group, respectively. However, it exhibited a significant decrease at 3, 12, and 24 h, with no statistically significant difference compared to the blank group (*p* = 0.2662) (Figure 3A).

After the digestion of the mantle, the distribution of Ca^2+^ within the mantle cells of the scallops was assessed using cellular Ca^2+^ imaging technology following a 1 h high-temperature treatment. The morphology of the mantle cells was observed under bright field microscopy, with nuclei stained by DAPI exhibiting blue fluorescence. Ca^2+^-positive signals labeled with Fluo-4 AM and Fluo-4FF were visualized as green fluorescence. Additionally, ER-tracker staining highlighted the ER, which displayed red fluorescence (Figure 3B). Quantitative analysis of the green Ca^2+^-positive signal revealed a significant increase in intracellular fluorescence intensity in the mantle cells after 1 h of high-temperature treatment, which was 4-fold that in the blank group (*p* < 0.05) (Figure 3C). Although there was a decrease in Ca^2+^-positive signals within the ER compared to the blank group, this change did not reach statistical significance (*p* = 0.0632) (Figure 3D). In the blank group, Fluo-4FF signals were predominantly localized to the endoplasmic reticulum. Upon high-temperature exposure, the Fluo-4 AM signal in the cytoplasm was intensified and broadly distributed, while the Fluo-4FF signal in the endoplasmic reticulum declined.

#### 2.3.2. *Py*RyR-like Domains Potentially Interact with *Py*PKA and *Py*FKBP12 In Vitro

**Figure 4:**
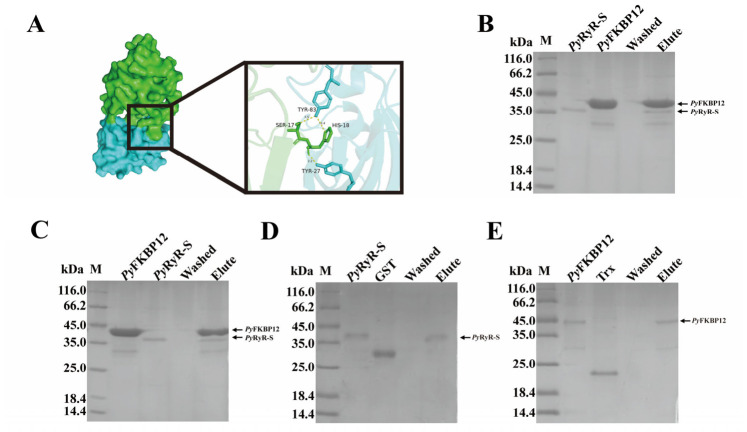
Interaction between r*Py*RyR-like-SPRY and r*Py*FKBP12: (**A**) Molecular docking analysis of *Py*RyR-like-SPRY and *Py*FKBP12. (**B**) Pull-down by r*Py*RyR-like-S (His). (**C**) Pull-down by r*Py*FKBP12 (GST). (**D**) Pull-down by rGST. (**E**) Pull-down by rTrx.

**Figure 5:**
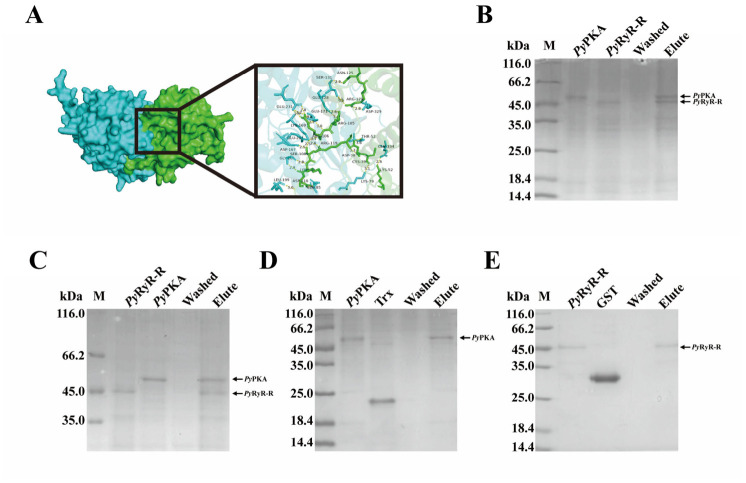
Interaction between r*Py*RyR-like-R domain and r*Py*PKA. (**A**) Molecular docking analysis of *Py*RyR-like-RYR and *Py*PKA. (**B**) Pull-down by rPyPKA (GST). (**C**) Pull-down by r*Py*RyR-like-R (His). (**D**) Pull-down by rTrx. (**E**) Pull-down by rGST.

AlphaFold3 was utilized to predict the three-dimensional structures of *Py*RyR-like-S, *Py*RyR-like-R, *Py*PKA, and *Py*FKBP12. To elucidate the potential binding mode between *Py*RyR-like-S and *Py*FKBP12, the presumed binding site of *Py*FKBP12 was first mapped onto the predicted structure of *Py*RyR-like-S. Docking grid boxes were then defined, and flexible docking was performed using AutoDock Vina, version 1.2.0. The interaction between *Py*PKA and *Py*RyR-like-R is analogous to that between *Py*FKBP12 and its counterpart. Specifically, the docking sites for the interaction between *Py*FKBP12 and *Py*RyR-like-S included residues S17 and H18. Meanwhile, the docking sites for *Py*PKA and *Py*RyR-like-R included D38, C39, K52, R105, R106, S108, K109, D110, R119, R124 and N125 (Figure 4A and Figure 5A).

r*Py*FKBP12, r*Py*RyR-like-S, r*Py*RyR-like-R and r*Py*PKA were expressed in *E. coli* transsetta (DE3) and purified using the His-tag and GST-tag protein fusion purification system. Distinct bands were observed by SDS-PAGE, consistent with the predicted molecular mass of r*Py*FKBP12 (44.8 kDa) (Figure S2A), r*Py*RyR-like-S (40.9 kDa) (Figure S2B), r*Py*RyR-like-R (45.6 kDa) (Figure S2C), and r*Py*PKA (54.4 kDa) (Figure S2D).

The purified r*Py*FKBP12 was subsequently utilized to generate a polyclonal antibody. The specificity of the anti-r*Py*FKBP12 antibody was assessed through lysate coating. Each channel exhibited a distinct band at approximately 12 kDa, which aligns with the predicted molecular weight of *Py*FKBP12 in relation to anti-*Py*FKBP12 (Figure S3A).

The interaction between r*Py*FKBP12 and r*Py*RyR-like-S, as well as r*Py*RyR-like-R and r*Py*PKA, was analyzed by the pull-down assay. There were two distinct bands corresponding to the protein-pair r*Py*FKBP12 and r*Py*RyR-like-S (Figure 4B,C) and the protein-pair r*Py*RyR-like-R and r*Py*PKA (Figure 5B,C) in the elute liquid after the pull-down assay. In the control group with rTrx-His or rGST as the bait protein to pull down r*Py*FKBP12, r*Py*RyR-like-S, r*Py*RyR-like-R and r*Py*PKA, only one band was observed in the elute liquid (Figure 4D,E and Figure 5D,E). Similarly, when r*Py*FKBP12, r*Py*RyR-like-S, r*Py*RyR-like-R and r*Py*PKA were used as the bait protein to pull down rTrx-His or rGST, only one band was observed in the elute liquid (Figure 4D,E and Figure 5D,E).

#### 2.3.3. High Temperature Induces Upr Activation and Apoptosis

**Figure 6:**
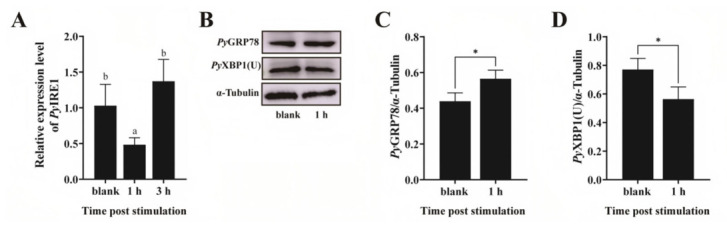
Expression level of UPR-related mRNA and protein after high-temperature treatment: (**A**) The relative expression level of *Py*IRE1 mRNA, and distinct letters (a and b) were used to indicate the significant differences. (**B**–**D**) Western blot analysis of *Py*GRP78 and *Py*XBP1(U) proteins. Mean ± S.D. is presented for each value with N = 4. * *p* < 0.05.

The relative expression levels of key UPR molecules were quantified to assess the activation of UPR in the mantle following high-temperature treatment. The relative expression of *Py*IRE1 mRNA decreased significantly at 1 h and recovered to a normal level at 3 h after high-temperature treatment (*p* < 0.05) (Figure 6A). Compared to the blank group, the protein level of *Py*GRP78 increased significantly by 1.29-fold after one hour of high-temperature treatment (*p* < 0.05) (Figure 6B,C). Conversely, the protein expression level of *Py*XBP1(U) decreased markedly to 0.73-fold of that in the blank group following one hour of high-temperature treatment (*p* < 0.05) (Figure 6B,D). 

**Figure 7:**
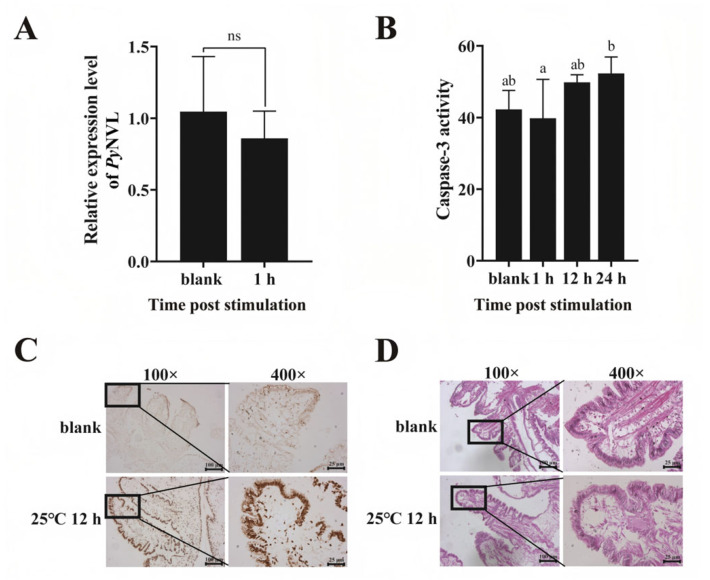
Changes in *Py*NVL mRNA expression, Caspase-3 activity and apoptosis after high-temperature treatment: (**A**) Relative expression level of *Py*NVL mRNA. (**B**) Caspase-3 activity. (**C**) TUNEL-stained mantle tissue section. (**D**) HE-stained mantle tissue section. Significant differences are indicated by distinct letters, and mean ± S.D. is presented for each value with N = 4. ns, not significant.

To detect the effect of temperature stress on the recovery of ER, the mRNA expression level of *Py*NVL was detected. After 1 h of high-temperature treatment, there was no significant change in the mRNA expression level of *Py*NVL compared with the blank group (*p* > 0.05) (Figure 7A).

To investigate the impact of high-temperature treatment on apoptosis and tissue damage, Caspase-3 activity was assessed, and tissue sections were subjected to HE staining as well as TUNEL analysis. Compared to the blank group, Caspase-3 activity exhibited a significant increase at 24 h, reaching 1.24-fold of that in the blank group (*p* < 0.05) (Figure 7B). TUNEL staining was utilized to assess apoptosis in cells subjected to high-temperature treatment. The nuclei of TUNEL-positive cells appeared dark brown. Compared with the blank group, there was a significant increase in the number of dark brown nuclei after 12 h of high-temperature treatment (Figure 7C). The HE staining results demonstrated that after 12 h of high-temperature treatment, the morphology and structure of the mantle tissue underwent significant changes (Figure 7D). Notably, there were prominent vacuoles observed between the connective tissues, muscle fibers exhibited signs of disruption, the number of nuclei was markedly reduced, and the original structural integrity was compromised (Figure 7D).

### 2.4. Dantrolene Treatment Alleviates High-Temperature-Induced UPR-Related Responses and Apoptosis in Scallop Mantle

#### 2.4.1. Dantrolene Reduces *Py*RyR-like/*Py*FKBP12 Expression and Cytoplasmic Ca^2+^ Accumulation Under High-Temperature Stress

**Figure 8:**
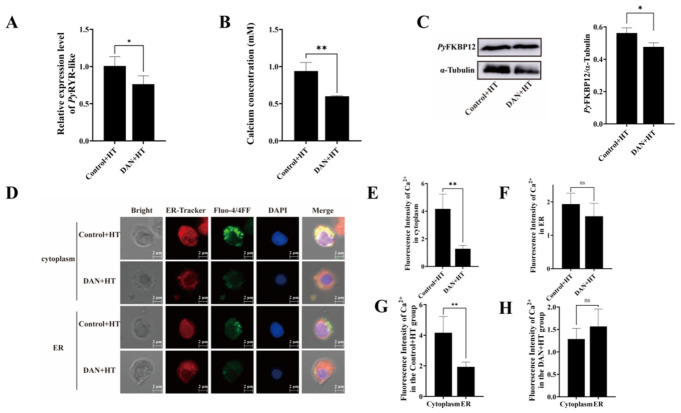
Changes in *Py*RyR-like mRNA expression, *Py*FKBP12 protein expression and Ca^2+^ concentration and distribution in scallop mantle after combined treatment of Dantrolene and high temperature: (**A**) Relative expression level of *Py*RyR-like mRNA. (**B**) The intracellular Ca^2+^ content. (**C**) Western blot analysis of *Py*FKBP12 protein. (**D**) The Ca^2+^ distribution. (**E**,**F**) The mean fluorescence intensity of Ca^2+^ in the cytoplasm and ER. (**G**,**H**) The mean fluorescence intensity of Ca^2+^ in the Control + HT group and the DAN+HT group. Each value was shown as mean ± S.D. (N = 4). * *p* < 0.05; ** *p* < 0.01; ns, not significant.

At 1 h after treatment, the relative expression of *Py*RyR-like mRNA (*p* < 0.05), concentration of Ca^2+^ (*p* < 0.01) and the protein expression level of *Py*FKBP12 (*p* < 0.05) in the mantle of the DAN + HT group were significantly reduced to 76%, 64% and 85% of the Control + HT group levels, respectively (Figure 8A–C).

The distribution of Ca^2+^ within the mantle cells of the scallops in the DAN + HT group was assessed at 1 h after treatment using cellular Ca^2+^ imaging technology. The nuclei stained by DAPI were shown as blue fluorescence, and Ca^2+^-positive signals labeled with Fluo-4 AM and Fluo-4FF were visualized as green fluorescence, while ER highlighted by ER-tracker staining was displayed as red fluorescence (Figure 8D). Quantitative analysis of the green Ca^2+^-positive signal revealed that the fluorescence intensity in the cytoplasm of the scallop in the DAN+HT group significantly decreased (0.31-fold of that in the Control + HT group, *p* < 0.01) (Figure 8E), while that within the ER showed no significant difference between the two groups (*p* > 0.05) (Figure 8F). Furthermore, in the Control + HT group, the Ca^2+^-positive signals in the cytoplasm were significantly higher than those in ER (2.15-fold, *p* < 0.01) (Figure 8G). However, the Ca^2+^-positive signals had no significant difference between the cytoplasm and ER in the DAN + HT group (*p* > 0.05) (Figure 8H).

#### 2.4.2. Dantrolene Suppresses High-Temperature-Induced Upr Activation and Apoptosis

**Figure 9:**
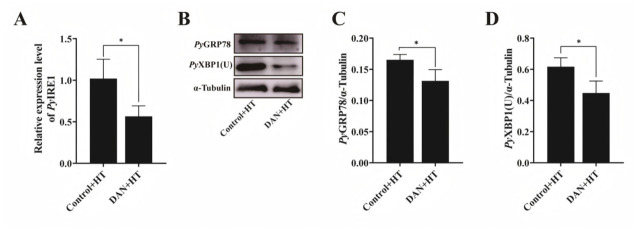
Expression level of UPR-related mRNA and protein after combined treatment of Dantrolene and high temperature: (**A**) The relative expression level of *Py*IRE1 mRNA. (**B**–**D**) Western blot analysis of *Py*GRP78 and *Py*XBP1(U) protein. Mean ± S.D. is presented for each value with N = 3. * *p* < 0.05.

The mRNA expression level of *Py*IRE1 in the DAN + HT group was significantly decreased at 1 h, which was 0.55-fold of that in the Control + HT group (*p* < 0.05) (Figure 9A). Similarly, the protein levels of *Py*GRP78 and *Py*XBP1(U) in the DAN + HT group were also significantly decreased, which were 0.85- and 0.73-fold of that in the Control + HT group, respectively (*p* < 0.05) (Figure 9B–D).

**Figure 10:**
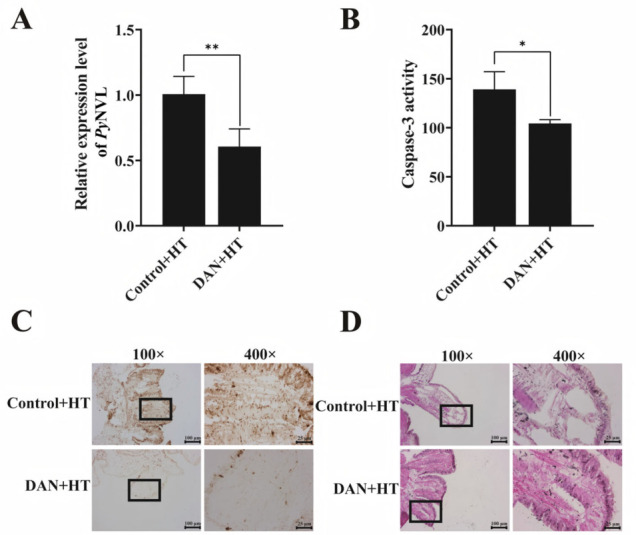
Changes in *Py*NVL mRNA expression, Caspase-3 activity and apoptosis after combined treatment of Dantrolene and high temperature: (**A**) Relative expression level of *Py*NVL. (**B**) Caspase-3 activity. (**C**) TUNEL-stained mantle tissue section. (**D**) HE-stained mantle tissue section. Mean ± S.D. is presented for each value with N = 4. * *p* < 0.05; ** *p* < 0.01.

Furthermore, the mRNA expression level of *Py*NVL (Figure 10A) and the activity of Caspase-3 (Figure 10B) in the DAN + HT group were significantly decreased at 1 h after treatment, which was 0.60-fold (*p* < 0.01) and 0.68-fold (*p* < 0.05) of that in the Control + HT group. Compared with the Control + HT group, the number of dark brown nuclei decreased significantly in the DAN + HT group (Figure 10C). Furthermore, changes in the morphology and structure of the mantle were analyzed by using HE staining. The Control + HT group showed obvious vacuoles and broken muscle fibers, while the connective tissue of the mantle in the DAN + HT group remained relatively intact and showed better original structural integrity (Figure 10D).

All the results in Section 3.4 indicated that Dantrolene exerts a protective effect on the mantle of scallops under high-temperature stress by inhibiting *Py*RyR-like, reducing cytoplasmic Ca^2+^ overload, alleviating ER stress and cell apoptosis, and mitigating tissue damage.

## 3. Discussion

### 3.1. Structural Conservation and Species Specificity of PyRyR-like

RyR-like is a Ca^2+^ release channel located in the ER membrane and plays an important role in maintaining calcium homeostasis in the intracellular environment [26,27,28,29,30,31,32,33]. In this study, a RyR-like consisting of 18 conserved structural domains was identified in the scallop *Patinopecten yessoensis* (*Py*RyR-like) and was highly conserved among RyR-like proteins from mammals and other aquatic species [4,5,34]. In addition to the typical structural domains, molecular binding sites interacting with *Py*RyR-like were identified on top of the structural domains, including 2 sites on SPRY1 interacting with *Py*FKBP12 and 11 sites on RYR3-4 interacting with *Py*PKA, which are key to controlling the opening of the channel, and these key functional sites exhibit a certain degree of evolutionary conservation and divergence relative to their counterparts in mammals and other aquatic organisms, suggesting that *Py*RyR-like can also be involved in a variety of functions similar to those of RyR-likes in other species [11,35,36]. In the evolutionary tree of RyR-likes in invertebrates and vertebrates, *Py*RyR-like first clusters with other molluscan RyR-likes, then with echinoderms, and finally with vertebrates, suggesting that molluscan RyRs are significantly different from their vertebrate homologs. In summary, *Py*RyR-like, a member of the RyR family in the scallop, retains conserved RyR family domains and may act as an important regulatory molecule for ER Ca^2+^ release. However, the relatively low sequence similarity between *Py*RyR-like and vertebrate RyR-like, together with its closer phylogenetic relationship with molluscan RyRs, suggests that *Py*RyR-like may also possess mollusk-specific structural and regulatory characteristics. Unlike mammals, scallops are cold-water marine bivalves with limited ability to escape acute environmental temperature fluctuations. Therefore, these structural differences may be associated with species-specific Ca^2+^ regulatory patterns, allowing scallops to adjust intracellular Ca^2+^ homeostasis and stress responses under high-temperature stress.

### 3.2. PyRyR-like-Related Ca^2+^ Regulation Under High Temperature Stress

Previous studies have shown that RyR channel density increases and expression is upregulated in response to temperature and different compound stimuli, suggesting that RyR is sensitive and easily activated by external stimuli [17,20,24]. Previous studies have shown that RyR channels in invertebrates such as *Placopecten magellanicus* and *Homarus americanus* display typical functional properties, including activation by Ca^2+^, caffeine, and ATP, as well as modulation by Mg^2+^ and ryanodine. In addition, studies on *Pecten jacobaeus* striated adductor muscle demonstrated that cADPR could trigger Ca^2+^ release through a ryanodine-sensitive channel. The outer membrane edge of shellfish mantle is rich in sensory cells, which can sensitively detect changes in water temperature and salinity. The cells also maintain body homeostasis by regulating ion transport, lipid metabolism, and signaling pathways to help shellfish respond and adapt to environmental stress. Similar regulatory patterns have been reported in previous studies. In *P. yessoensis*, TRPA1-like has been shown to regulate UPR and apoptosis by mediating Ca^2+^ influx under high-temperature stress, suggesting that Ca^2+^ disturbance is closely associated with ER stress-related responses and apoptosis in scallops [37]. In addition, in human umbilical vein endothelial cells, intense heat stress was reported to elevate cytoplasmic Ca^2+^ levels and induce apoptosis through a Ca^2+^-mediated mitochondrial apoptotic pathway, accompanied by the activation of caspase-9 and caspase-3 [38]. In this study, high temperature significantly upregulated the mRNA expression of *Py*RyR-like, while Dantrolene treatment reduced this response. These results suggest that *Py*RyR-like may be involved in the high temperature response of scallop mantle. FKBP12, which is essential for stabilizing RyR channels, was upregulated under temperature domestication, and the depletion of FKBP12 resulted in the loss of the SR calcium store, which was mainly achieved by altering channel gating [39,40,41]. After being activated by cAMP, PKA binds to the RyR-like complex through anchoring proteins, catalyzing phosphorylation of key serine sites in RyR-like, which subsequently causes conformational changes in RyR-like, reducing its binding affinity to FKBP12.6, promoting dissociation of the PKA-FKBP-RyR-like complex, increasing the probability of RyR-like channel opening, and mediating endoplasmic reticulum Ca^2+^ release [9,42]. In the present study, High-temperature was able to significantly upregulate the protein expression level of *Py*FKBP12, whereas Dantrolene was able to inhibit this effect, which is another evidence that *Py*RyR-like can be activated by high temperature. Although AlphaFold-based prediction and pull-down assays support the possible interaction between *Py*RyR-like and *Py*FKBP12/*Py*PKA, these results mainly reflect in vitro binding. Further in vivo evidence will be needed to confirm the formation of the *Py*RyR-like-*Py*FKBP12-*Py*PKA complex in scallop cells.

Many studies have shown that RyRs play an important role in maintaining calcium homeostasis and helping cells to perform normal physiological activities under environmental stress conditions [43,44]. In this study, cytoplasmic Ca^2+^ content was significantly increased 1 h after heat induction and significantly decreased 1 h after Dantrolene and heat treatment. Ca^2+^ released by RyR was significantly increased in the cells after heat stress, whereas the increase was significantly suppressed after Dantrolene treatment, suggesting that RyR plays a major role in Ca^2+^ release from the cytosolic SR, consistent with previous observations [45]. ER Ca^2+^ did not show significant changes after high-temperature stress and inhibitor treatment, which may be related to SERCA, a calcium pump on the ER, and involves the mechanism of ER calcium uptake (cardiac excitation–contraction coupling) [46]. ER stress affects Ca^2+^ release; particularly, accumulation of unfolded proteins in the ER leads to Ca^2+^ leakage into the cytoplasm [47]. These findings suggest that *Py*RyR-like may participate in intracellular Ca^2+^ redistribution under high-temperature stress, especially in the increase in cytosolic Ca^2+^ signals. However, Fluo-4 AM and Fluo-4FF differ in Ca^2+^ affinity, loading efficiency and subcellular localization; therefore, the fluorescence patterns in Figure 3E,F should be interpreted mainly as relative distribution changes in the two probes under the same imaging conditions.

### 3.3. Relationship Among Ca^2+^ Disturbance, UPR-Related Responses and Apoptosis

The ER is the main storage organelle for Ca^2+^ and the source of intracellular Ca^2+^ signaling [47]. RyR can mediate the release of Ca^2+^ from the ER to the cytoplasm, thereby altering ER calcium homeostasis and triggering ER stress [48]. When ER stress occurs, GRP78 first responds and activates the IRE1-XBP1 pathway, leading to the activation of the ER-associated degradation (ERAD) mechanism, which in turn removes unfolded or misfolded proteins to restore ER homeostasis [49]. Dantrolene partially inhibited zinc-induced elevation of intracellular Ca^2+^ levels, suggesting that RyR is involved in ER stress due to disturbed ER Ca^2+^ homeostasis [50]. In this study, high temperature led to ER stress, which was mainly reflected by the changes in *Py*GRP78 and *Py*XBP1(U), two important molecules involved in UPR activation. *Py*NVL was further detected in order to preliminarily evaluate the possible recovery of ER after stress.

Apoptosis will be triggered after prolonged ER stress or excessive stress. Oxidative stress induces ER stress, activates the IRE1/XBP1 pathway, and promotes the expression of the apoptotic marker Caspase-3 [50]. Dantrolene treatment decreased cleaved Caspase-3 apoptosis protein and increased pro-survival protein kinase C (PKC) protein levels, suggesting a role for RyRs in mediating calcium imbalance [51]. Consistent with previous studies, in the present study, caspase-3 activity increased significantly 24 h after 25 °C treatment, indicating that *Py*RyR-like may be involved in the regulation of Caspase-3 through the release of Ca^2+^, thus promoting apoptosis under high-temperature stress. This was further supported by the corresponding number of positive apoptotic cell nuclei observed under TUNEL staining. In conclusion, high temperature can trigger ER stress and apoptosis, and 25 °C high-temperature treatments can activate *Py*RyR-like channels, cause ER calcium release, and alter the calcium homeostasis of the intracellular environment, thus leading to ER stress and cell apoptosis, indicating that *Py*RyR-like plays an important role as a Ca^2+^ channel in the high-temperature stress response of scallops. These findings contribute to a deeper understanding of the function of *Py*RyR-like in maintaining Ca^2+^ homeostasis, regulating UPR and apoptosis in scallops under high-temperature stress. Based on research in mammals, heat stress induced calcium dyshomeostasis and subsequently triggered ER stress-mediated apoptosis, supporting the close relationship between stress-induced Ca^2+^ disturbance and ER stress activation [52]. Another study further demonstrated in a mammalian model that Ca^2+^ imbalance, ER stress, and RyR-like-related signaling are tightly coupled, highlighting the important role of RyR-like-associated calcium dysregulation in stress injury [53]. These studies are consistent with our findings that high temperature activated the *Py*RyR-like-*Py*FKBP12-*Py*PKA axis, increased intracellular Ca^2+^ levels, and was accompanied by UPR-related responses in scallop mantle. In studies on other aquatic animals, for instance, studies on *Macrophthalmus japonicus* [54] and *Fundulus heteroclitus* [55] have demonstrated that the expression of RyR-like can be altered in response to external stimuli, indicating that the regulatory pattern of *Py*RyR-like conforms to the general molecular mechanism underlying high-temperature stress response in shellfish. In this study, the possible involvement of the IRE1-XBP1 pathway under heat stress was inferred from the combined changes in *Py*GRP78, *Py*IRE1 and *Py*XBP1(U). However, because the splicing status of XBP1 was not directly examined, the present results are still insufficient to fully characterize the activation pattern of the IRE1-XBP1 pathway. In the present study, high-temperature treatment significantly increased the protein level of *Py*GRP78 and decreased the level of *Py*XBP1(U), suggesting that an ER stress-related UPR occurred in the scallop mantle. Meanwhile, *Py*XBP1(S) showed an increasing trend after high-temperature treatment. The anti-XBP1(S) antibody used in this study produced a single detectable band in the scallop mantle samples. The apparent molecular weight of this band was close to the predicted size of XBP1(S), indicating that this antibody could recognize a scallop XBP1(S)-like protein to some extent. In addition, the Dantrolene treatment reduced the levels of *Py*GRP78, *Py*XBP1(U), and *Py*XBP1(S), further suggesting that RyR-related Ca^2+^ disturbance was associated with the UPR under high-temperature stress. In addition, the downregulation of *Py*IRE1 mRNA may reflect a negative feedback regulation after ER stress rather than suppression of the pathway. However, apoptosis appeared to be a relatively late response to heat stress, as Caspase-3 activity increased significantly at 24 h, whereas intracellular Ca^2+^ change and redistribution were more evident at the early stage, especially at 1 h. Therefore, 1 h was selected for the combined treatment experiment to focus on the early role of *Py*RyR-like in Ca^2+^ regulation. However, the lack of later validation remains a limitation of this study.

### 3.4. Limitations and Future Perspectives

A limitation of the present study is that *Py*RyR-like activation was not directly verified by electrophysiological recording. Owing to the technical limitations of current bivalve cell systems, patch-clamp analysis was not feasible. Therefore, the possible involvement of *Py*RyR-like was inferred from early coordinated changes in cAMP, *Py*PKA activity, *Py*RyR-like/*Py*FKBP12 expression, intracellular Ca^2+^ signal, and the inhibitory effect of Dantrolene. Second, *Py*NVL was used in this study to preliminarily evaluate the possible recovery status of the ER after high temperature stress. However, since only the mRNA expression level of *Py*NVL was determined, the evidence for ER recovery remains limited. Therefore, more direct evidence, such as *Py*NVL protein detection or analysis of additional ER-related components, will be needed in future studies to further clarify the recovery status of the ER under high temperature stress. Third, although extracellular Ca^2+^ influx cannot be completely excluded, calcium-free buffer was used during imaging preparation to minimize this contribution, suggesting that the observed increase in cytoplasmic Ca^2+^ was mainly associated with intracellular Ca^2+^ mobilization. Fourth, because the anti-XBP1(S) antibody used in this study was developed against mammalian XBP1(S), the interpretation of PyXBP1(S) results in scallop should be cautious. Although this antibody produced a single detectable band with an apparent molecular weight close to the predicted size of XBP1(S), further validation of its cross-reactivity and direct detection of *Py*XBP1(S)-related molecular changes are still needed. These additional studies will help clarify the role of the IRE1-XBP1 branch in the response of scallops to high temperature stress.

**Figure 11:**
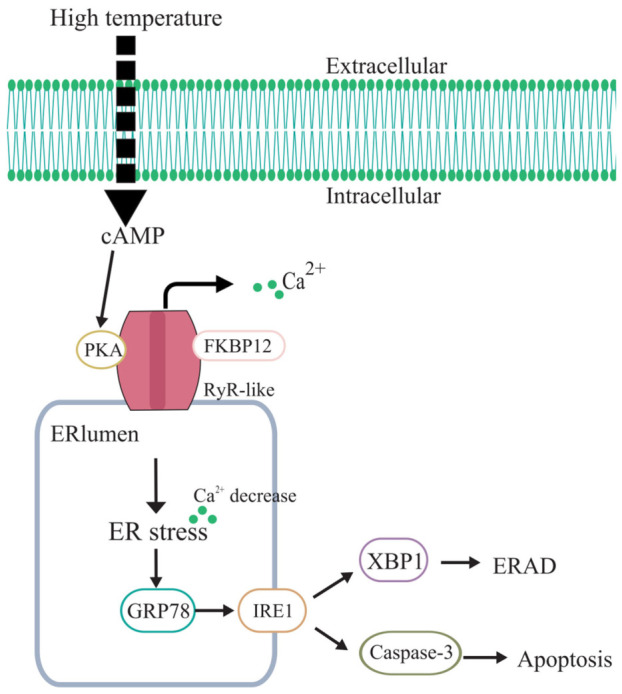
Schematic diagram of the regulation mechanisms of *Py*RyR-like under high-temperature stress.

In conclusion, a *Py*RyR-like with classical structural characteristics was identified from scallops and its tissue distribution was clarified. Furthermore, its molecular mechanism in response to high temperature suggests that the *Py*RyR-like-*Py*FKBP12-*Py*PKA complex regulates intracellular Ca^2+^ in scallops under high temperature(25 °C), and elucidates the stress response pathway of *Py*RyR-like mediating IRE1α-UPR and apoptosis through regulating calcium homeostasis. These findings provide new insight into the molecular mechanism underlying scallop adaptation to high temperature (Figure 11).

## 4. Materials and Methods

### 4.1. Scallops, Treatments and Sample Preparation

#### 4.1.1. Scallop

Two-year-old Yesso scallops were collected from Zhangzidao aquaculture farm in Dalian, China [37]. Before the experiment, the scallops were acclimated in aerated natural seawater at 15 ± 1 °C for 7 days. During acclimation, the scallops were fed with spirulina powder once daily, and the seawater was completely renewed once daily to maintain water quality. All the experiments were conducted in accordance with the guidelines established by the ethics review committee and with consent from Dalian Ocean University [37].

Hemocytes and tissues including gill, hepatopancreas, adductor muscle, mantle and gonad were extracted and preserved for subsequent experiments following an established protocol [37].

#### 4.1.2. High-Temperature Treatment

The scallops were temporarily acclimated in seawater at 15 °C for one week, ensuring that the water was changed and the scallops were fed daily. In total 120 scallops were transferred to seawater at 25 °C and subjected to treatment durations of 1, 3, 6, 12 and 24 h, respectively. The remaining 24 scallops kept at 15 °C seawater were employed as the blank group. Mantle was collected for RNA extraction, protein extraction, and analysis of *Py*PKA activity, cAMP levels, and calcium content.

The time points of 1, 3, 6, 12 and 24 h were selected based on previous acute high-temperature stress studies in *P. yessoensis*, in which Ca^2+^-related signals, UPR-related genes and metabolic indicators showed rapid responses within 1–6 h and recovery, attenuation or secondary changes at 12–24 h [37].

#### 4.1.3. Combined Treatment with Ryr Antagonist and High Temperature

In total 24 scallops were randomly divided into two groups (*n* = 12 per group), namely the vehicle control group and the Dantrolene treatment group. The scallops in the Dantrolene treatment group were pretreated with 200 μL of 600 μM Dantrolene per scallop (dissolved in a vehicle consisting of 10% DMSO, 40% PEG300, 5% Tween-80, and 45% saline). The scallops in the vehicle control group received an equal volume of the same vehicle without Dantrolene. At 24 h after injection, the scallops in both groups were exposed to seawater at 25 °C for 1 h, and the samples were named the DAN + HT group and the Control + HT group, respectively. Mantle tissues were collected for RNA extraction, protein extraction, calcium content analysis, and immunohistochemistry.

### 4.2. The cDNA Synthesis, Gene Cloning and Sequence Analysis

#### 4.2.1. RNA Isolation and cDNA Synthesis

Total RNA was extracted from mantle tissues using an RNA extraction reagent according to the manufacturer’s instructions, with minor modifications. Briefly, mantle tissues were homogenized on ice and centrifuged at 12,000× *g* for 5 min at 4 °C. The supernatant was mixed with chloroform substitute, vortexed thoroughly, and centrifuged at 12,000× *g* for 15 min at 4 °C. The upper aqueous phase was transferred to a new RNase-free tube and mixed with an equal volume of pre-cooled isopropanol to precipitate RNA at −80 °C for 60 min. After centrifugation, the RNA pellet was washed twice with 75% ethanol, air-dried, and dissolved in DEPC-treated water. RNA quality and concentration were assessed using agarose gel electrophoresis and a NanoDrop spectrophotometer. First-strand cDNA was synthesized using the TransScript® one-step gDNA removal and cDNA synthesis kit according to the manufacturer’s instructions. The reverse transcription reaction was performed in a 20 μL reaction mixture containing total RNA, Anchored Oligo(dT)18 primer, 2 × TS Reaction Mix, TransScript® RT/RI Enzyme Mix, and gDNA Remover. The reaction was carried out at 42 °C for 15 min, followed by 85 °C for 5 s. The synthesized cDNA was diluted 10-fold with nuclease-free water and stored at −80 °C for subsequent PCR and qRT-PCR analyses.

#### 4.2.2. Cloning of *Py*RyR-like-Spry Domain, *Py*RyR-like-Ryr Domain, *Py*FKBP12 and *Py*PKA

The primers for cloning the SPRY and RYR domains of *Py*RyR-like, *Py*PKA and *Py*FKBP12 were designed based on the sequence information of *Py*RyR-like (XM_021514914.1), *Py*PKA (XM_021510196.1) and *Py*FKBP12 (XM_021483747.1), respectively (Table 1). PCR amplification was performed using TaKaRa Ex Taq DNA polymerase in a 50 μL reaction system containing 0.25 μL TaKaRa Ex Taq (5 U/μL), 5 μL 10 × Ex Taq Buffer, 4 μL dNTP mixture, 2 μL cDNA template, 2 μL forward primer (10 μM), 2 μL reverse primer (10 μM), and 34.75 μL nuclease-free water. PCR amplification was performed under the following conditions: 94 °C for 3 min; 35 cycles of 94 °C for 30 s, annealing at 5 °C below the Tm value of each primer pair for 30 s, and 72 °C for 1 min per kb; followed by a final extension at 72 °C for 10 min. The PCR products were examined by agarose gel electrophoresis, purified, cloned into the pMD19-T simple vector (TaKaRa), and verified by DNA sequencing. 

#### 4.2.3. Sequence Analysis of *Py*RyR-like

The conserved domains were predicted using SMART (http://smart.embl-heidelberg.de/, accessed on 15 June 2026). The multi-sequence alignment and the NJ phylogenetic tree construction were performed using MEGA X, version 10.2.6. The Bootstrap test was performed 1000 times to obtain confidence values for the phylogenetic analysis [56]. All sequence information is described in Table 2.

### 4.3. Quantitative Reverse Transcription Pcr (Qrt-Pcr) Analysis

SYBR Green qRT-PCR was performed on an ABI 7500 real-time PCR system using a TaKaRa SYBR Green qPCR kit according to the manufacturer’s instructions. The primers used for qRT-PCR are listed in Table 1. Each reaction was performed in a 10 μL reaction system containing SYBR Green mix, ROX reference dye, diluted cDNA template, forward and reverse primers, and RNase-free water. The qRT-PCR program was as follows: 95 °C for 30 s, followed by 40 cycles of 95 °C for 5 s and 60 °C for 34 s. A melting curve analysis was performed at the end of amplification to confirm the specificity of the PCR products. qRT-PCR was performed with four biological replicates, and each biological replicate was analyzed with three technical replicates.

*Py*EF-α was used as the internal reference gene. It was selected based on a previous reference-gene evaluation in *P. yessoensis* under high-temperature treatment, in which PyEF-α showed high expression stability in mantle tissues [57]. The relative mRNA expression levels of *Py*RyR-like, *Py*IRE1 and *Py*NVL were calculated using the 2^−ΔΔCt^ method [58,59].

### 4.4. Recombinant Protein and Polyclonal Antibody Preparation

Recombinant proteins of the *Py*RyR-like-SPRY domain, *Py*RyR-like-RYR domain, *Py*PKA, and *Py*FKBP12 (these recombinant proteins were named as r*Py*RyR-S, r*Py*RyR-R, r*Py*PKA and r*Py*FKBP12, respectively) were expressed in *Escherichia coli* and purified as previously described with some modification [58,60]. The target fragments were amplified by PCR using specific primers listed in Table 1. After digestion with the corresponding restriction enzymes, the fragments were ligated into pET-30a or pCold-GST expression vectors using T4 DNA ligase. The recombinant plasmids were transformed into *Escherichia coli* Transetta (DE3) cells for protein expression. Positive transformants were cultured overnight in LB medium at 37 °C with shaking. The overnight culture was then inoculated into fresh LB medium and grown at 37 °C. Recombinant protein expression was induced by adding IPTG to a final concentration of 1 mmol/L. His-tagged recombinant proteins were purified using a His-Tag Protein Purification Kit, while GST-tagged recombinant proteins were purified using GST Sepharose 4FF according to the manufacturers’ instructions. The purified recombinant proteins were analyzed by SDS-PAGE and stored at −80 °C for subsequent experiments.

To prepare the polyclonal antibody against *Py*FKBP12, purified r*Py*FKBP12 was used to immunize 6-week-old mice. The specificity of the anti-*Py*FKBP12 antibody was verified by Western blot before use.

### 4.5. Western Blot Assays

Total protein from the mantle of the scallops after high-temperature treatment was subjected to Western blot assays according to the previous description. The anti-GRP78 (AF0171, Beyotime, Shanghai, China, 1:1000), anti-XBP1(U) (AF8367, Beyotime, Shanghai, China, 1:1000), anti-XBP1(S) (A22546, Abclonal, Wuhan, China, 1:1000), and anti-FKBP12 (1:500) were used as primary antibodies, and HRP-labeled goat anti-rabbit IgG at a concentration of 1:1000 was used as a secondary antibody. After blocking at room temperature for 3 h, the membranes were incubated with primary antibodies at 4 °C overnight. After washing with TBST, the membranes were incubated with HRP-labeled goat anti-rabbit IgG at room temperature for 3 h. The specificity of all the commercial antibodies was detected through Western blot. The signal was developed using an automated chemiluminescence gel imaging system (Amersham Imager 600, GE Healthcare Life Sciences, Marlborough, MA, USA). α-Tubulin was used as the loading control, and band intensities were normalized to α-Tubulin.

### 4.6. Detection of Camp Concentration, Pypka Activity and Calcium Content

#### 4.6.1. Detection of Camp Concentration

The cAMP concentration in the mantle was measured using a cAMP ELISA Kit (D770001-0096, Sangon Biotech, Shanghai, China) according to the manufacturer’s instructions. The tissue samples were mixed with 0.1 M HCl at a volume ratio of 5–10:1 (HCl: sample) and homogenized on ice. The homogenate was then centrifuged at 600× *g* for 5 min at room temperature, and the supernatant was collected for analysis. Standard wells, sample wells, B0 wells, and non-specific binding (NSB) wells were prepared. Neutralizing Buffer (50 µL) was added to all the wells, and 50 µL of Sample Diluent was added specifically to the NSB and B0 wells. Subsequently, 50 µL of standard solution and sample solution were added to their corresponding wells, followed by the addition of 50 µL of Assay Buffer to the NSB wells. Afterward, 50 µL of cAMP-HRP Conjugate was added to all the wells, and 50 µL of anti-cAMP monoclonal antibody was added to all the wells except the NSB wells. The plate was incubated at room temperature for 2 h and then washed four times with Assay Buffer. Next, 150 µL of TMB substrate was added to each well and incubated at room temperature for approximately 10 min. The reaction was terminated by adding 50 µL of Stop Solution to each well, and the absorbance was measured at 450 nm using a microplate reader. The cAMP concentration was calculated based on the standard curve.

#### 4.6.2. Detection of Pypka Activity

*Py*PKA activity in the mantle of the scallops after high-temperature treatment was assessed using the PKA Colorimetric Activity Kit (EIAPKA, Invitrogen, Carlsbad, CA, USA) following the manufacturer’s instructions. For every 100 mg of mantle tissue, 400 µL of lysis buffer containing Cell Lysis Buffer, protease inhibitor, PMSF, and sodium orthovanadate (at a volume ratio of 1000:1:1000:500) was added, and the tissue was homogenized on ice. The homogenate was incubated for 30 min on ice and then used for analysis. Standard and sample wells were prepared by adding 40 µL of either standard solution or sample to each well, followed by 10 µL of reconstituted ATP. After sealing the plate, it was incubated on a shaker at 30 °C for 90 min. The wells were then emptied, washed four times with 300 µL of washing buffer, and tapped dry. Next, 25 µL of donkey anti-rabbit IgG HRP conjugate and Phospho-PKA Substrate Antibody mixture was added to each well and incubated at room temperature for 1 h with gentle shaking. The wells were again washed four times with washing buffer, and 100 µL of TMB substrate solution was added to each well and incubated at room temperature for 30 min. The reaction was stopped by adding 50 µL of Stop Solution to each well, and absorbance was measured at 450 nm using a microplate reader. *Py*PKA activity was calculated from the standard curve.

#### 4.6.3. Detection of Calcium Content

Calcium content in the mantle was determined using a calcium colorimetric assay kit (S1063S, Beyotime Biotechnology, Shanghai, China) according to the manufacturer’s instructions and previously described protocols [61]. The mantle was collected, homogenized in an appropriate buffer on ice, and centrifuged to obtain the supernatant. Calcium concentration was then determined colorimetrically using a microplate reader.

### 4.7. Immunofluorescence Assay

To investigate the intracellular distribution of calcium in the mantle cells, the calcium-sensitive fluorescent probes Fluo-4 AM (5 µM) and Fluo-4FF (10 µM) were employed. Several modifications were made based on previously established protocols [55,62].

The mantle tissues were carefully cut into 2 mm square pieces, washed twice with PBS, and digested in 0.05% Trypsin-EDTA solution at 37 °C for approximately 1 h. The resulting cell suspension was passed through a cell strainer, and approximately 2 mL of resuspension buffer was added before centrifugation at 600× *g* for 10 min at 4 °C. The supernatant was discarded, and the washing step was repeated twice. The cells were then resuspended in 200–400 µL of buffer and seeded onto slides, allowing them to settle at 4 °C for 1 h. According to previous reports, the cells were incubated successively with Fluo-4 AM and Fluo-4FF, ER-Tracker and DAPI [63].

### 4.8. Interaction Between PyRyR-like-S, PyRyR-like-R, Pypka and Pyfkbp12

#### 4.8.1. Structural Prediction of *Py*RyR-like-S, *Py*RyR-like-R, *Py*PKA and *Py*FKBP12 Protein Using Alphafold3

The three-dimensional structures of the *Py*RyR-like-S, *Py*RyR-like-R, *Py*PKA, and *Py*FKBP12 proteins were predicted using the AlphaFold3 server (https://alphafoldserver.com/, accessed on 15 June 2026) [64]. The amino acid sequences of the target proteins were submitted to the AlphaFold3 prediction pipeline, which integrates multiple sequence alignments (MSA) generated by MMseqs2 with advanced deep learning models. All the predictions were conducted using default parameters [64]. The reliability of the predicted modules was assessed based on the predicted Local Distance Difference Test (pLDDT) score [65]. According to the AlphaFold confidence criteria, regions with pLDDT > 90 were considered highly confident, regions with 70 < pLDDT ≤ 90 were considered confident, regions with 50 < pLDDT ≤ 70 were considered to have low confidence, and regions with pLDDT ≤ 50 were considered very low confidence. The predicted models with acceptable confidence were selected for subsequent structural visualization and interaction analysis. The visualization and structural analysis were performed using PyMOL, version 2.6.2 (http://pymol.sourceforge.net/, accessed on 15 June 2026) to reveal key structural features and domains [66].

#### 4.8.2. Pull-Down Assay

A pull-down assay between r*Py*RyR-like-R (His tag) and r*Py*PKA (GST tag), r*Py*RyR-like-S (His tag) and r*Py*FKBP12 (GST tag) was further conducted. r*Py*RyR-like-R and r*Py*PKA, r*Py*RyR-like-S and r*Py*FKBP12 were added to 2 mL of charged Ni-NTA beads (for His-tagged proteins) and incubated at room temperature with gentle rotation for 4 h. The mixture (resin and binding proteins) was washed three times with PBS by centrifugation at 200× *g* for 2 min to eliminate the unbound proteins. Subsequently, the proteins from the test tissues were added to the mixture and incubated with gentle rotation at room temperature for another 4 h. After the resin was washed three times, the bound proteins were separated by 12.5% SDS-PAGE. rTrx-His (His) and rGST pull-down assays for r*Py*RyR-like-R and r*Py*PKA, r*Py*RyR-like-S and r*Py*FKBP12 were carried out as controls, respectively.

### 4.9. Detection of Apoptosis

#### 4.9.1. Caspase-3 Activity Analysis

After high-temperature treatment and inhibitor treatment, the Caspase-3 activity in the mantle was determined using the Caspase-3 activity assay kit (C1116, Beyotime) as previously described [55].

#### 4.9.2. Histological Analysis

The mantle was fixed in Bouin’s Fluid for 24 h, subsequently dehydrated through a graded ethanol series (75%, 80%, 85%, 90%, and 100%), rendered transparent with xylene, and embedded in paraffin. Sections (4 µm thick) were prepared and affixed to microscope slides, followed by dewaxing with xylene and rehydration through a graded ethanol series. All the sections were stained with Hematoxylin and Eosin Staining Kit following the manufacturer’s protocol (Beyotime).

#### 4.9.3. Tunel Assay

Apoptosis was assessed using a TUNEL kit (C1098, Beyotime, Shanghai, China) in accordance with the manufacturer’s protocol [67]. Dehydrated paraffin sections were incubated with protease K at 37 °C for 30 min and washed three times with PBS, and then incubated with an Endogenous Peroxidase Blocking Buffer at room temperature for 20 min, followed by three washes with PBS. The reaction mixture of the TUNEL reagent was applied at 37 °C for 60 min in the dark. Following the PBS washes, the DAB chromogenic solution was utilized, with brown-stained cells indicating positive TUNEL staining.

### 4.10. Statistical Analysis

All data are presented as mean ± standard deviation (SD). For all the animal experiments, each treatment group contained six scallops (*n* = 6) as biological replicates. For cell-based assays, including calcium imaging and immunofluorescence, hemocytes from three scallops were pooled to prepare one mixed-cell sample. For molecular assays, qRT-PCR was performed with four independent biological replicates (*n* = 4), while Western blot and pull-down assays were each performed with three independent biological replicates (*n* = 3). Statistical comparisons between two groups were conducted using the independent-samples Student’s *t*-test, whereas comparisons among multiple groups were performed using one-way analysis of variance (ANOVA). Differences were considered statistically significant at *p* < 0.05 and highly significant at *p* < 0.01.

## Figures and Tables

**Table 1:** Sequences of the primers used in the present study.

Primer Name	Primer Sequences (5’-3’)
**qRT-PCR primer**	
*Py*RyR-like-RTF	GCGTGAGAAGGATATCGCCA
*Py*RyR-like-RTR	TACTGGTCAGCATAACGGGC
*Py*IRE1-RTF	AGCCAGTTCAGATGGCATGA
*Py*IRE1-RTR	CAAGTGAGGTCAAGGCAAAGT
*Py*NVL-RTF	ACAGTGTTCGGGAGGACCTA
*Py*NVL-RTR	GAAGAGTTCCTAGCCCGCTG
*Py*EF*-α*-RTF	GCGGTGGTATTGACAAGAGA
*Py*EF*-α*-RTR	GTTCACGTTCAGCCTTCAGT
**Cloning primer**	
*Py*RyR-like-SPRY-F	GGAGTAGCAGTGAGAACCAACC
*Py*RyR-like-SPRY-R	CTGTTCTATCTTGTTCATGGCCC
*Py*RyR-like-RYR-F	CTGCCATTGGTTGTGCTCTG
*Py*RyR-like-RYR-R	CCGTCGTGAGAGTTACAGCA
*Py*PKA-F	TCACCCTCAACAGAACACAGCC
*Py*PKA-R	CCAGAACTCCTAAAGCCCACCA
*Py*FKBP12-F	AGTTTGCCCCGAAGGAATAA
*Py*FKBP12-R	TCCAGTGCTGCGGTGAATG
**Recombinant primer**	
*Py*RyR-like-SPRY-MQ-F	CGGGATCCGGAGTAGCAGTGAGAACCAA
*Py*RyR-like-SPRY-MQ-R	CCCAAGCTTCTGTTCTATCTTGTTCATGGC
*Py*RyR-like-RYR-MQ-F	CGGGATCCGCCATTGGTTGTGCTCTGTCT
*Py*RyR-like-RYR-MQ-R	CCCAAGCTTGCTGTAACTCTCACGACGGGA
*Py*PKA-MQ-F	GGGGTACCCACCCTCAACAGAACACAGCC
*Py*PKA-MQ-R	AACTGCAGCAGAACTCCTAAAGCCCACCA
*Py*FKBP12-MQ-F	CGGAATTCATGGGAGTAACGAAAAAAAC
*Py*FKBP12-MQ-R	AACTGCAGTCACTTGAGTCTAATAAGTTCTACA

**Table 2:** Sequences used for the *Py*RyR-like alignment and phylogenetic analysis.

Protein Name	Orgainism	Accession Number
RyR2	*Homo sapiens*	NP_001026.2
RyR2	*Mus musculus*	NP_076357.2
RyR2	*Danio rerio*	XP_068080773.1
RyR2	*Oryctolagus cuniculus*	NP_001076226.1
RyR2	*Rhineura floridana*	XP_061480564.1
RyR2	*Octopus bimaculoides*	XP_052827807.1
RyR2	*Holothuria leucospilota*	KAJ8044966.1
RyR2	*Strongylocentrotus purpuratus*	XP_030855111.1
RyR2	*Mytilus galloprovincialis*	VDI80221.1
RyR2-L	*Ruditapes philippinarum*	XP_060571016.1
*Py*RyR-like	*Patinopecten yessoensis*	XP_021370589.1
RyR2-L	*Saccostrea echinata*	XP_061163617.1
RyR2	*Caenorhabditis elegans*	NP_001343688.1
RyR	*Drosophila melanogaster*	NP_001246209.1

## Data Availability

Data will be made available on request.

## Supplementary Materials

The following supporting information can be downloaded at https://www.mdpi.com/article/10.3390/ijms27135859/s1.
