# Supplementary figures and images for: The RyR-like-FKBP12-PKA Complex Regulates Intracellular Ca2+, Unfolded Protein Response and Apoptosis in Patinopecten yessoensis Under High-Temperature Stress
Source: Int J Mol Sci. 2026 Jun 29;27(13):5859. doi: 10.3390/ijms27135859 (PMC13362341; doi:10.3390/ijms27135859)

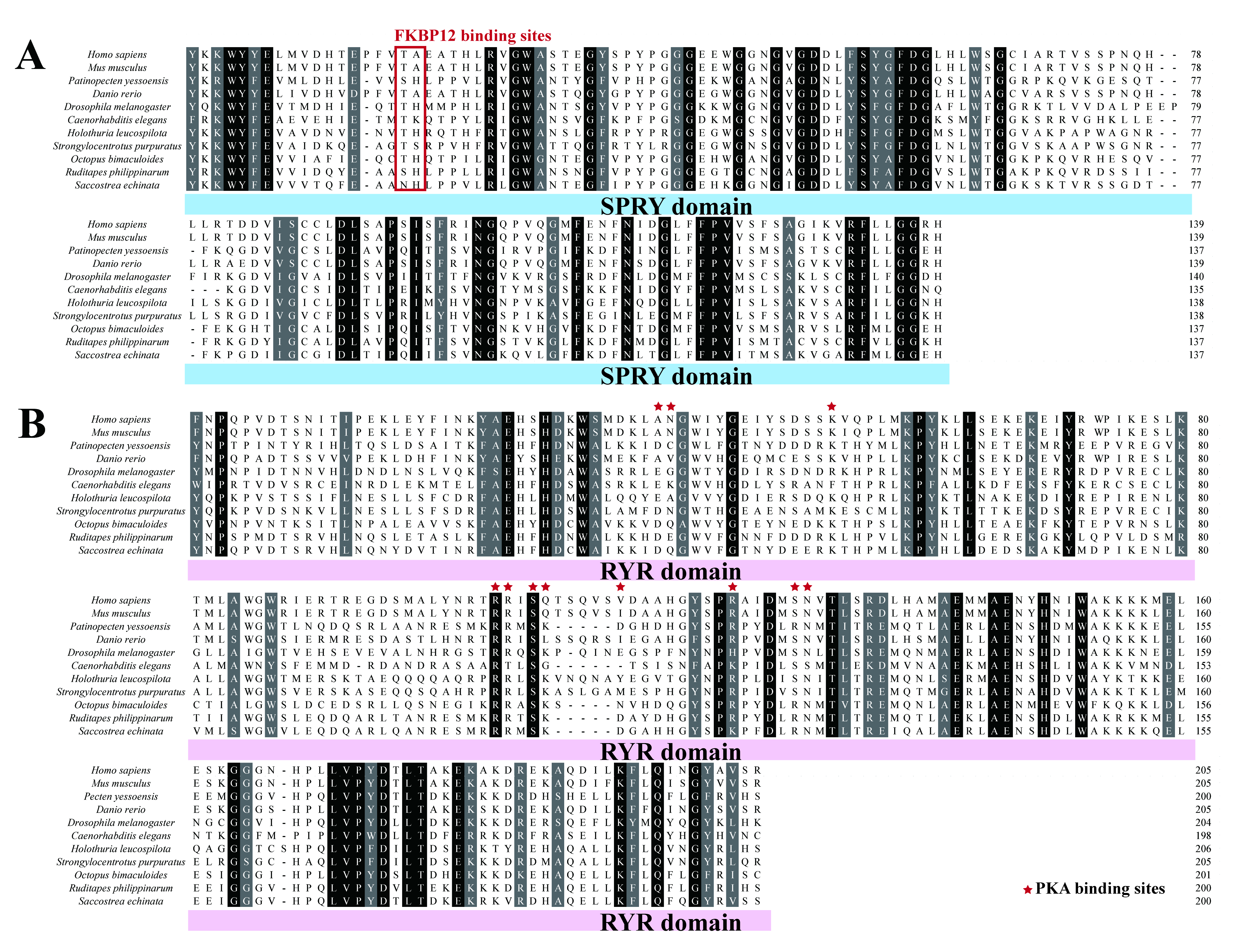

Supplement: Supplementary file 1 [file ijms-27-05859-s001.zip › Fig S1.tif]

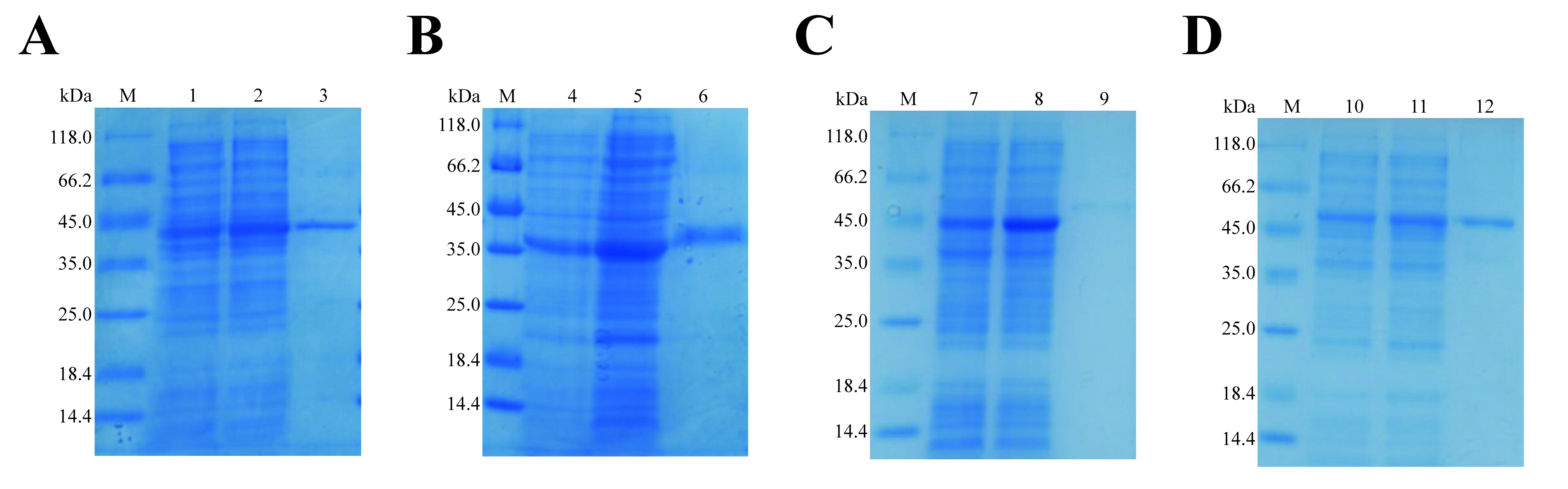

Supplement: Supplementary file 1 [file ijms-27-05859-s001.zip › Fig S2.tif]

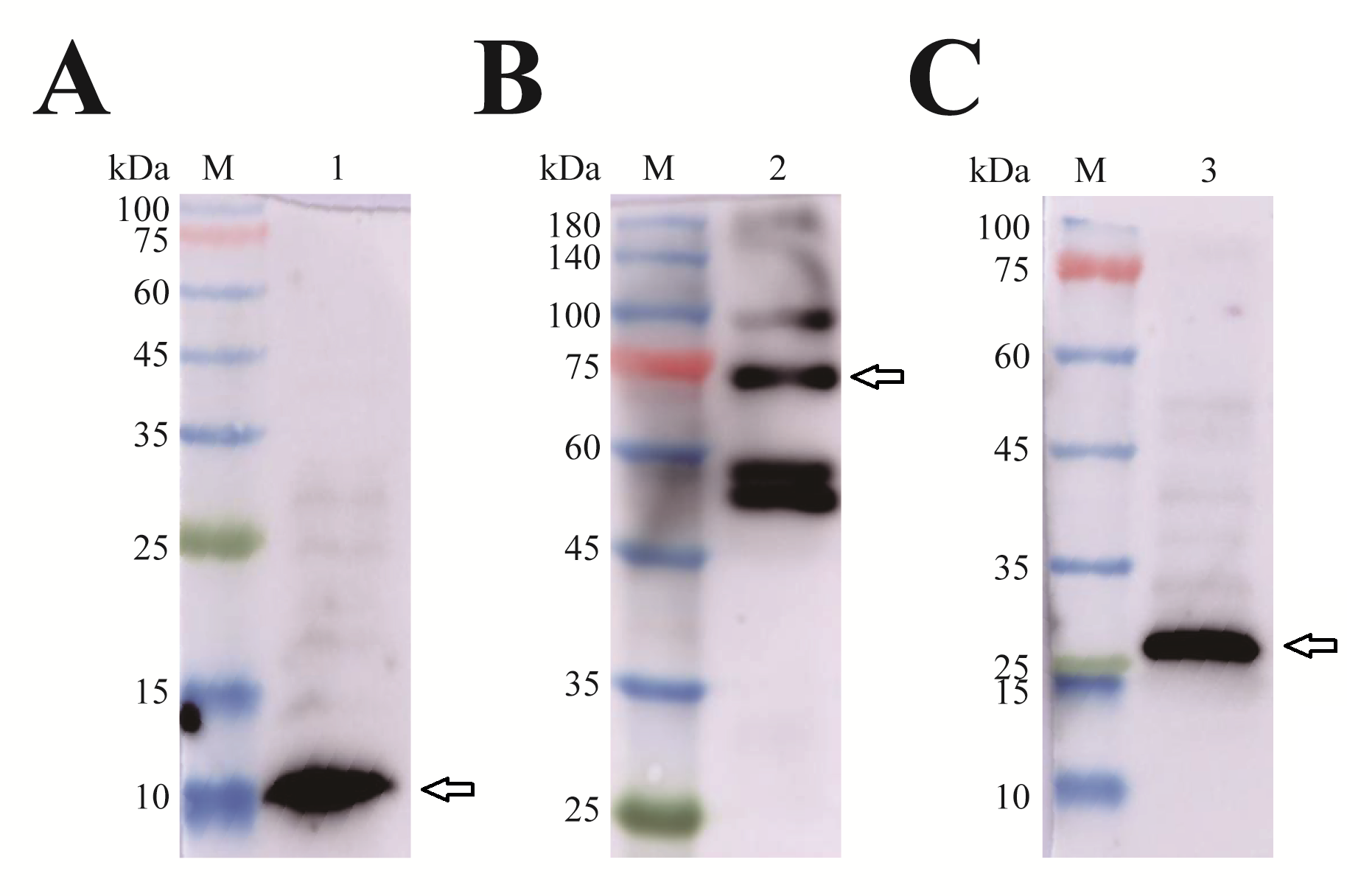

Supplement: Supplementary file 1 [file ijms-27-05859-s001.zip › Fig S3.tif]
